# Evaluating the dentistry program in Iran using the context, input, process, and product (CIPP) model: a comprehensive analysis

**DOI:** 10.3389/fmed.2024.1394395

**Published:** 2024-07-19

**Authors:** Fatemeh Babadi, Misagh Esfandiari, Maria Cheraghi

**Affiliations:** ^1^Department of Oral and Maxillofacial Medicine, School of Dentistry, Ahvaz Jundishapur University of Medical Sciences, Ahvaz, Iran; ^2^Social Determinants of Health Research Center, Ahvaz Jundishapur University of Medical Sciences, Ahvaz, Iran; ^3^Department of Public Health, School of Health, Ahvaz Jundishapur University of Medical Sciences, Ahvaz, Iran

**Keywords:** curriculum, dentistry, CIPP, Ahvaz, Iran

## Abstract

**Introduction:**

Attaining a commendable level of quality in an educational program is not just important but imperative. Hence, this study was undertaken to assess the quality of the general dentistry program for students in Iran, utilizing the comprehensive Context, Input, Process, and Product (CIPP) evaluation model.

**Methods:**

This cross-sectional study was carried out among dentistry students in the 5th to 13th semesters at Ahvaz Jundishapur University of Medical Sciences in Ahvaz, Iran. Data collection for this research utilized a questionnaire designed in alignment with the CIPP Evaluation Model. The perspectives of students were sought in assessing the four domains of context, input, process, and product. Statistical analysis of the data was conducted using ANOVA and *T*-test methods.

**Results:**

The mean scores of the educational program were as fallow: Content (2.76 ± 0.58), input (2.71 ± 0.65), process (2.51 ± 0.68), and product (3.31 ± 0.68). Overall, the quality of dentistry program was undesirable in all dimensions. Among these dimensions, “product” had the highest mean, while “process” had the lowest mean score.

**Conclusion:**

The results of this study indicate that the general dentistry educational program were Undesirable in all domains. The CIPP evaluation framework assists decision-makers and policymakers in determining the continuation or renewal of a training program by identifying its strengths and weaknesses.

## Introduction

Reviewing the curriculum of universities is a continuous, necessary, and unavoidable phenomenon. Like other scientific fields, dentistry is also influenced by external factors and the development of interdisciplinary advancements (1). Therefore, it is necessary to update its curriculum in line with scientific and environmental changes. Therefore, it is necessary to update the curriculum in line with the scientific advancements and environmental changes. On the other hand, reviewing medical education is inevitable due to its responsiveness to global trends in healthcare, new technologies, emerging diseases, new patient expectations, the explosion of knowledge, and the increase in information about the human body (2).

Research indicates that dental graduates have reported that their skills and abilities are not up to the desired level (3). For example, the study conducted by Razavi et al. (1) demonstrated that courses in anatomical sciences and oral health and society had the highest alignment with the job requirements of dentists, while courses in parasitology and biostatistics had the lowest alignment.

Evaluation of the dentistry curriculum can be done from various perspectives. One of the important sources in determining the objectives of curriculum programs is the learner, who should have the content of the programs aligned with their needs (4, 5). One of the very important and effective solutions in identifying the quality of clinical education is to examine the opinions of the dentistry student (5).

The CIPP evaluation model (Context, Input, Process, Product), a management-oriented evaluation model, facilitates program evaluation throughout and following implementation across four dimensions: context, input, process, and output (6).

The purpose of evaluating the context is to provide a logical framework for determining educational goals. It also involves analytical efforts to identify relevant elements in the learning environment and to identify problems, needs, and opportunities in a context or educational situation. Input evaluation helps in designing and selecting appropriate methods to achieve the goals (7). Process evaluation is carried out to identify or predict implementation problems in the course of educational activities and to assess the desirability of the process of implementing these activities Product evaluation is conducted to judge the effectiveness of educational activities (8). In fact, the results of the program are compared with the program’s goals, and the relationship between expectations and actual results is determined.

A study conducted in 2021 by Rashidi Meybodi et al. (9) Conducted to examine the quality of periodontics educational program in Yazd University of Medical Sciences using the CIPP model. The results of this study indicated that in the periodontics department, input, process and product were undesirable for the students. Based on the above information, it seems that the dental curriculum in Iran should be evaluated and assessed to prevent a decline in global quality standards and irreparable damage to health. So, the aim of this study was to assessment of quality of the general course of dentistry program using the CIPP model as one of the most important and widely used models for evaluation from the view point of the students and then examine the relationship of its dimensions with demographic variables.

## Materials and methods

### Population and sampling

The present study is a cross-sectional study conducted at the School of Dentistry, Jundishapur University of Medical Sciences in Ahvaz, IRAN in 2023. The target population of this study was all dentistry students in 7th to 13th semesters at Ahvaz Jundishapur University of Medical sciences. Sampling was done by using census sampling method. The inclusion criteria for the study were being a fifth to thirteenth-semester dentistry student, willingness to participate in the study, and completing the informed consent. The exclusion criteria were non-Iranian students who do not have proficiency in the Persian language and who incomplete questionnaire completion.

### Measurement

The data collection was after the study approved by the Ethics Committee in Ahvaz Jundishapur University of Medical Sciences (Ethics Code: IR.AJUMS.REC.1402.498).

### Standard CIPP evaluation model questionnaire

The questionnaire above has been designed and validated by Mahshid AbdiShahshahani et.al based on the CIPP model for evaluating various educational courses. This questionnaire consisted of two parts. The first part included demographic information such as age, gender, academic semester, grade point average (GPA), and level of interest in the field of study. The second part of the questionnaire consisted of 156 questions in 4 domains: context, input, process, and output. To evaluate the responses, A 5-point Likert scale was used from very high, high, moderate, low, to very low and the range is calculated by (5 - 1). The total scores were calculated and divided by the number of questions. If the final score for each domain was less than 3.7, the status of that domain was considered undesirable; if it was between 3.7 and 4, the status was weak, and if it was between 4 and 5, the status was moderate, and above 5, the status was considered desirable. To assess the validity of the questionnaires, formal and content validity were used, and in terms of the alignment of the questionnaire items with the research topic and objectives, experts confirmed the use of Cronbach’s alpha coefficient, with values estimated at (0.98, 0.96, 0.98, 0.98), respectively (6).

### Data analysis

Descriptive tables were used to analyze the data, with frequency and percentage indices for qualitative variables, and mean and standard deviation indices for quantitative variables. The distribution of data (normality) was examined using the Kolmogorov-Smirnov test. Independent t-tests and analysis of variance tests were used to examine the mean scores of the questionnaire with demographic variables. Descriptive tables and statistical analysis were performed using SPSS software version. The statistical significance level in this study was set at 0.05.

## Results

A total of 245 participants were included in the study. 160 participants were men (65.3%) and 85 participants were women (34.7%). More than half of the participants were 24 years old or younger (58.8%), while And the other participants, 101 participant (41.2%) were over the 24 years old. The distribution of participants based on their grade point average Students were measured GPA was as follows: only 24 individuals (9.8%) had a GPA of 18 or higher, and nearly half of them (53.3%) had a GPA between 17.9 and 16. Regarding the level of interest in the academic field of study, 148 participants (60%) had a high or very high level of interest, only 21 participants (8.6%) had a low level of interest, and the remaining participants had an average level of interest in their the academic field of study (Table 1).

**TABLE 1:** Demographic and background characteristics of participants (*n* = 245).

Variable	Frequency	Percentage	Cumulative frequency	Cumulative frequency percentage
**Gender**				
Male	160	65.30	160	65.30
Female	85	34.70	245	100
**Age**				
22–20	71	29.00	71	29.00
22.1–24	73	29.80	144	58.80
24.1–26	50	20.40	197	79.20
26.1<	51	20.80	245	100
**Semester**				
5	37	15.10	37	15.10
7	53	21.60	90	36.70
9	66	26.90	156	63.60
11	56	22.90	212	86.50
13	33	13.50	245	100
**GPA**				
12–15.99	89	36.30	245	100
16–17.99	132	53.90	156	63.70
18≤	24	9.80	24	9.80
**Level of interest in the academic field of study**				
Low	21	8.60	21	8.60
Moderate	76	31.00	97	39.60
High	112	45.70	209	85.30
Very high	36	14.70	245	100

In Table 2, the Mean (SD) of the four dimensions of CIPP model, including context, input, process, and output, are shown. Overall, the program quality in this study was reported as undesirable in all dimensions. Among these dimensions, “output” had the highest average, while “ ” had the lowest average.

**TABLE 2:** Mean and SD of course’s evaluation dimensions based on CIPP model.

Dimensions	Mean	Standard deviation
Context	2.76	0.58
Input	2.71	0.65
Process	2.51	0.68
Output	3.31	0.68

According to the results of Table 3, a significant difference was observed in the mean score of output dimension among male and female dentistry students participating in the study. Although the output score is undesirable in both age groups, from the perspective of female compared to males, Jundi Shapur Ahvaz University of Medical Sciences has been more successful in achieving the desired effectiveness of the general dentistry education programs (outputs) (*P* = 0.020).

**TABLE 3:** Mean and SD of CIPP model’s dimensions based on gender difference.

Dimensions	Male	Female	t^#^	*P*-value
Context	2.77 ± 0.58	2.75 ± 0.59	0.203	0.840
Input	2.77 ± 0.67	2.59 ± 0.61	1.968	0.050
Process	2.49 ± 0.73	2.54 ± 0.59	-0.651	0.516
Output	3.25 ± 0.66	3.47 ± 0.71	-2.234	0.020*

^#^Independent sample *t*-test,

*P-value < 0.05.

Based on the results of Table 4, in the context dimension, fifth-semester students had significantly different opinions compared to ninth, eleventh, and thirteenth-semester students, considering the university more successful in the context of the general dentistry education program. Seventh-semester students also reported higher mean score in the context dimension compared to ninth and eleventh-semester students (*P* = 0.05). In the input dimension, fifth-semester students reported higher mean score compared to ninth and eleventh-semester students.

**TABLE 4:** Mean and SD of CIPP model’s dimensions based on semesters.

Dimensions	5-semester	7-semester	9-semester	11-semester	13-semester	T^#^	*P*-value
Context	3.05 ± 0.53	2.94 ± 0.52	**2.68 ± 0.56^be^**	**2.53 ± 0.57^cf^**	**2.73 ± 0.63^d^**	6.325	0.001**
Input	2.99 ± 0.64	2.78 ± 0.55	**2.66 ± 0.64^b^**	**2.50 ± 0.70^cf^**	2.72 ± 0.66	3.540	0.008**
Process	2.77 ± 0.61	2.80 ± 0.59	**2.37 ± 0.66^be^**	**2.29 ± 0.73^cf^**	**2.39 ± 0.64^dg^**	6.736	0.001**
Output	3.54 ± 0.65	3.33 ± 0.63	3.34 ± 0.63	**3.22 ± 0.78^c^**	3.23 ± 0.72	1.394	0.237

^#^One-Way ANOVA (“analysis of variance”). Shows significant difference between measurements (*P* < 0.05). a: significant difference between 5 and 7 Semesters, b: significant difference between 5 and 9 Semesters, c: significant difference between 5 and 11 Semesters, d: significant difference between 5 and 13 Semesters, e: significant difference between 7 and 9 Semesters, f: significant difference between 7 and 11 Semesters. *P*-value for significant results are shown in bold.

*P-value < 0.05, **P-value < 0.001.

Based on the results of Table 5 from the perspective of students who had a GPA of 18 and above, the Jundishapur Ahvaz University of Medical Sciences has been more successful in achieving the desirability of the educational programs of the general dental course (output) compared to the other two GPA groups (*P* = 0.012).

**TABLE 5:** Mean and SD of CIPP model’s dimensions based on GPA.

Dimensions	≥18	16–17.99	14–15.99	t^#^	*P*-value
Context	2.91 ± 0.52	2.77 ± 0.58	2.72 ± 0.61	1.001	0.369
Input	2.87 ± 0.68	2.68 ± 0.64	2.69 ± 0.67	0.896	0.409
Process	2.88 ± 0.81	2.49 ± 0.59	2.42 ± 0.75	4.514	0.012*
Output	3.28 ± 0.69	**3.38 ± 0.73^b^**	**3.26 ± 0.61^a^**	0.819	0.442

^#^One-Way ANOVA (“analysis of variance”). Shows significant difference between measurements (*P* < 0.05). a: significant difference between “≥18” and “14–15.99”: significant difference between “≥18” and “16–17.99”. *P*-value for significant results are shown in bold.

*P-value < 0.05.

## Discussion

Based on our research, the present study is the first paper on the educational status of the general dentistry program at Jundishapur Ahvaz University of Medical Sciences. The findings of this research indicate that the general dentistry educational program faced difficulties in achieving its educational objectives in all areas and had undesirable quality in all dimensions during.

According to the findings of the present study, the quality of the educational program was undesirable in the context dimension. More than 50% of the students mentioned the student-to-teacher ratio, insufficient clinical departments and operating rooms, lack of student rest areas, inadequate computer systems, lack of appropriate nutrition facilities in the faculty, inadequate financial budget, and unsuitable educational materials as reasons for the poor quality of the educational context. The results of this study were inconsistent with the findings of Makarem et al. (10) and Rashidi et al. (9). In the study by Rashidi et al. (9), the quality of educational program was examined in periodontics and oral health departments at Yazd University of Medical Sciences, It was demonstrated in the study that the context quality was relatively desirable in the periodontics and oral health departments. Furthermore, according to the findings of the present study, in the research conducted by Jafari et al. (11) at the School of Dentistry in Tehran University of Medical Sciences, students expressed dissatisfaction with the lack of sufficient educational equipment, inadequate human resources, and insufficient provision of adequate spaces.

One possible reason for this difference could be that most students of Ahvaz University of Medical Sciences have achieved higher rankings in the national entrance exam compared to Yazd University of Medical Sciences, and therefore have higher expectations from the accepted university. Another important reason is the relatively limited physical space of the dental school, which is evident in the related responses regarding the lack of suitable practice rooms, insufficient space for the school’s cafeteria, insufficient space for clinical departments, and lack of dedicated spaces for students. It seems that allocating more space to the dental school is necessary to address this issue. It is strongly recommended to organize courses for utilizing educational videos as another solution to enhance the quality of educational program context.

Based on the findings of the present study, the input was considered undesirable. The analysis of the results showed that, the content and educational objectives, educational facilities, offered courses, sequence and logical connection between the courses, computer and library facilities were all considered undesirable. One of the most important problems in the field of internal data was that 60% of the students identified problems in the lack of compatibility between the number of students and the “educational and recreational facilities” and the “sports and recreational facilities”. It seems better to reconsider the increase in the capacity of dental fields or allocate more financial resources to provide educational, cultural, and welfare facilities. In the study by Tabari et al. (12), the analysis of the results showed that, the content and educational objectives, educational facilities, the number of attendees (patients), the number of professors, the skills of professors, and the supervision of students’ performance were desirable. In the study by Makarem et al. (10), the quality of intraoperative education programs in the periodontics department and in the social oral health and dentistry department was relatively satisfactory, which contradicted the present study. It is possible that due to the presentation of a high volume of content in the curriculum, theory-based education may not be consistent with practical needs, and this lack of coherence may be the reason. On the other hand, differences in the treatment protocols used by professors in different groups, as well as the lack of alignment of some treatment with the protocols provided in the references, can be reasons for students’ acquired perspectives. Despite these potential shortcomings, further investigation is needed to find possible solutions and address them.

Based on the findings of the present study, the process dimension were considered undesirable. The evaluation of the process is carried out in order to identify or predict executive problems in the course of educational activities and the desirability of the implementation process of these activities. In the present study, the lack of importance given to student opinions in planning, the lack of welfare facilities in the department, and the mismatch between the number of students and the physical space were among the most important areas of low-quality processes. One of the factors evaluated in the scope of the process was the use of innovative teaching methods for better learning of learners and the use of new clinical training facilities to improve the educational level, which according to 56% of students, innovative teaching methods were not used, and according to 58% of students, new training facilities were not utilized. In contrast to the present study, Jafari et al. (11) reported relatively desirable quality scores for educational program processes. Similarly, in Rashidi Meybodi et al.’s study (9), in the oral health group of Yazd University of Medical Sciences, all four areas were of desirable quality.

From the perspective of students, the weaknesses that existed in the product were: the lack of teamwork among students and professors, inappropriate feedback from university officials in clinical education, inappropriate customer satisfaction and orientation, and inappropriate cost reduction and efficiency. Alongside the present study, the quality of the education program at Mashhad University of Medical Sciences was also undesirable (10). A qualitative study by Kham Verdi et al. (13) at Hamadan University of Medical Sciences on graduates of the general dental program showed that the achievement of educational goals in theoretical education for the restorative group was desirable, which contradicted the results of the present study. In the research by AliMohammadi et al.(14) Conducted at Rafsanjan Medical School, the product was relatively evaluated as desirable, which was not consistent with the results of the present study. It seems necessary to adopt strategies to improve the educational experience of students and ensure appropriate and desirable interaction between officials and students.

Based on the findings of the present study, from the perspective of female students compared to males, Jundishapur Ahvaz University of Medical Sciences has been more successful in achieving the desirability of the product of general dentistry education program. This study is in contrast to the study by Rashidi Meybodi et al. (15), in which no significant difference was observed between the opinions of female and male students in the four domains of CIPP in the periodontics course. In the study by Zamanzad et al. (16), it was also reported that gender did not play a significant role in the satisfaction with the quality of clinical course education at Shahrekord University of Medical Sciences. It seems that discovering the possible causes of these differences requires further investigation, but it is possible that these differences are due to societal expectations and culture, which impose greater stress on males to achieving professional success{Rafatjah, 2012 #163}. Therefore, these stress may be the reasons for these differences.

Furthermore, the present study has shown that eleventh-semester students have the least satisfaction in all dimensions except for the process compared to students 1 year below themselves. The findings of the present study were consistent with Rashidi et al. (9) Study, as in that study, both groups of periodontics and oral health departments reported that eleventh-semester students have the least satisfaction. It seems that the increase in awareness and insight of students in higher semester, as well as their interest in continuous learning and their stress for entering the job market, has created a general dissatisfaction among this group of students; while the level of satisfaction may increase slightly in the thirteenth semester due to the acquisition of more skills and experience.

According to the findings of the present study, there was a significant difference in the output dimension of the general dental education program among students with different GPAs. In other words, from the perspective of students who had a GPA of 18 and above, Jundishapur Ahvaz University of Medical Sciences has been more successful in achieving the desirability output of the educational program for general dental education. Very few studies have considered the important variable of students’ GPA in evaluating their quality of the educational program. Based on the research team’s searches, only in the study of Mirzaei Alavijeh et al{Mirzaei-Alavijeh, 2021 #165}, had examined this variable, and showed a relationship between high GPA and better evaluation of the educational program process. It seems that this relationship indicates the importance of appropriate assessment methods in training and the significance of considering students’ opinions in implementing the desired educational programs.

## Conclusion

Despite the 2012 updates aimed at tackling issues like overcrowding and limited elective courses in the dental curriculum, student perception suggests that the desired quality remains elusive. The latest revisions appear to have fallen short in boosting student satisfaction. The presence of dynamic young faculty members and adequate resources lays the foundation for progress. Acknowledging these findings and engaging stakeholders, particularly students, can amplify effectiveness and drive educational program enhancements for overall improvement.

## Data availability statement

The raw data supporting the conclusions of this article will be made available by the authors, without undue reservation.

## Ethics statement

The studies involving humans were approved by the Ethics Committee in Ahvaz Jundishapur University of Medical Sciences (Ethics Code: IR.AJUMS.REC.1402.498). The studies were conducted in accordance with the local legislation and institutional requirements. Written informed consent for participation in this study was provided by the participants’ legal guardians/next of kin.

## Author contributions

FB: Supervision, Writing – review and editing. ME: Project administration, Writing – original draft. MC: Conceptualization, Formal analysis, Supervision, Writing – review and editing.

## References

[1] Rezaei Z Ramazanzade K Abbaszadeh H. The degree of compliance of the content of the general dentistry curriculum with job needs from the perspective of dentists in Birjand. *Iran J Med Educ.* (2021) 21:68–81.

[2] Ahangari Z Rahmani M Sohrabi Z Kharazifard M. Dental Curriculum According to Views of the Graduates of Dental Schools of the Country During the Last 5 Years. *Shahid Beheshti Univ Dental J.* (2010) 28:80–87.

[3] Nejad Shamsi P Zaker-Jafari H Basirat M Zaker-Jafari A. Self-assessment of senior dental students about acquired skills based on the educational program. *Res Med Educ.* (2017) 9:78–73. 10.29252/rme.9.3.79

[4] Khoshrang H Salari A Dadgaran I Moaddab F Rouh-Balasii L Pourkazemi I. Quality of education provided at the clinical skills lab from medical students’ viewpoints in Guilan University of Medical Sciences. *Res Med Educ.* (2016) 8:77–83. 10.18869/acadpub.rme.8.2.77 30186993

[5] Babaee N Jahanian I Bijani A Ardebili Haghighi MR. Viewpoints of dental students about practical value of educational contents in oral medicine department. *Med Educ J.* (2014) 2:35–40.

[6] AbdiShahshahani M Ehsanpour S Yamani N Kohan S Hamidfar B. The evaluation of reproductive health PhD program in Iran: a CIPP model approach. *Proc Soc Behav Sci.* (2015) 197:88–97. 10.1016/j.sbspro.2015.07.059

[7] Rachmaniar R Yahya M Lamada M. Evaluation of learning through work practices industry program at University with the CIPP model approach. *Int J Environ Eng Educ.* (2021) 3:59–68. 10.55151/ijeedu.v3i2.55

[8] Riyad MK Wang Y Pakarinen J. Measuring the professionality of school teachers’ performance: the context, input, process, and product (CIPP) model’. *Int J Innov Creativ Change.* (2020) 12:386–99.

[9] Rashidi Maybodi F Hosseini-Yekani A Golshahi N. Assessment of educational status of periodontics and oral health and community dentistry departments in Yazd dental school using CIPP model in 2018. *JSSU*. (2021) 29:4046–56.

[10] Makarem A Movahed T Sarabadani J Shakeri MT Asadian Lalimi T Eslami N. Evaluation of educational status of oral health and community dentistry department at Mashhad Dental School using CIPP evaluation model in 2013. *J Mashhad Dent Sch.* (2014) 38:347–62.

[11] Jafari A Khami M Yazdani R Mohamadi M. Presenting the course of community dentistry as problem based learning workshop and comparing it to learning through lecture. *Iran J Med Educ.* (2010) 9:216–24.

[12] Tabari M Nourali Z Khafri S Gharekhani S Jahanian I. Evaluation of educational programs of pediatrics, orthodontics and restorative departments of babol dental school from the perspective of the students based on the CIPP model. *Caspian J Dent Res.* (2016) 5:8–16.

[13] Khamverdi Z Kasraee S Rostamzadeh T Yekta H. Educational objectives achieved by department of operative dentistry: viewpoints of general dentists graduated from Hamedan dental school (2004-2009). *Iran J Med Educ.* (2012) 12:387–95.

[14] Alimohammadi T Rezaeian M Bakhshi H VaziriNejad R. The evaluation of the Medical School Faculty of Rafsanjan University of Medical Sciences based on the CIPP model in 2010. *J Rafsanjan Univ Med Sci.* (2013) 12:205–18.

[15] Rashidi Maybodi F Hosseini-Yekani A Golshahi N. Assessment of educational status of periodontics and oral health and community dentistry departments in yazd dental school using CIPP model in 2018. *J Shahid Sadoughi Univ Med Sci.* (2021) 29:4046–56. 10.18502/ssu.v29i8.7665

[16] Zamanzad B Moezi M Shirzad H. *Rate of Satisfaction and Evaluation of Medical Students (Interns and Externs) from the Quality of Clinical Education in the Shahre-Kord University of Medical Sciences-2005.* (2007) 9:13–20.

